# Educational Psychology-Empowered Creative Practice Strategy and Educational Countermeasures for Cinematography Major

**DOI:** 10.3389/fpsyg.2022.913294

**Published:** 2022-07-04

**Authors:** Hanwei Di

**Affiliations:** School of Theatre Film & Television, Communication University of China, Beijing, China

**Keywords:** film studies, educational psychology, creative skills, theoretical knowledge, educational countermeasures

## Abstract

This work aims to improve Cinematography Majors’ creative orientation and practical skills and improve related teaching quality. Firstly, this work analyzes the theoretical knowledge and main working principles of Educational Psychology (EPSY). Then, it reviews the current situation and characteristics of the Cinematography teaching through a Questionnaire Survey (QS). Consequently, an EPSY-based teaching effect evaluation model is proposed for Cinematography Majors. The results show that genders have great differences in Cinematography Majors’ theoretical knowledge and creative orientation. Girls’ theoretical knowledge learning effect is better than boys, with about 84% qualification rate at best. Boys’ creative orientation learning effect is better than girls, with the highest qualification rate of about 84%. Meanwhile, students’ theoretical knowledge differs greatly from grade to grade and the learning effect increase with the grade. Nevertheless, students’ overall creative orientation is not satisfactory. Lastly, students’ theoretical knowledge differs greatly given different artistic backgrounds, but the difference in creative orientations is small. Thus, the school can carry out targeted teaching for students according to different genders, grades, and artistic backgrounds, to comprehensively improve the teaching effect of Cinematography. The finding provides technical support and educational countermeasures for improving the teaching effect of Cinematography and the reform of Cinematography teaching.

## Introduction

With the development of the times, the educational sector has become the main driving force for social development and thus become the top priority in many countries (Machatschek and Lendlein, 2020). Cinematography plays an important role in the development of the film industry. Therefore, it is necessary to study and improve the teaching strategies of a Cinematography Major. Besides, in developing an innovative society, cultivating Cinematography Majors’ innovation and entrepreneurship ability is also essential (Bell et al., 2019). Although the innovative research of cinematography is still immature, many studies have provided technical support for further exploration.

Klevan (2020) argued that film literary criticism has shown a diversified trend since the new century. Miscellaneous criticism methods enriched film literary criticism activities and promoted the development of literary film creation. However, while making achievements, there were also some problems. For example, the weakening of the criticism standard of the unity of historical and esthetic views was replaced by the separation and even opposition between historical views and esthetic views. Such views have deviated from the original meaning of the classical methodology of Marxist literary and artistic research in the specific interpretation (Klevan, 2020). Georgiadou and Kolaxizis (2019) reasoned that investigating the problems in the film industry was based on the problems in education. Therefore, teaching reform could comprehensively improve the problems in the Cinematography teaching and the film industry (Georgiadou and Kolaxizis, 2019). Graesser et al. (2022) observed that in the recent 20 years, China’s Educational Psychology (EPSY) has been unprecedentedly prosperous. The discipline system has been perfected, but its research results have promoted the reform and development of China’s education in many aspects. The main manifestations were as follows. First, leaps and bounds have been made in discipline system construction, research field expansion, and the Sinicization of research orientation. Second, EPSY provided a theoretical basis for quality education. Third, EPSY has laid the psychological science foundation for curriculum teaching reform. Fourth, EPSY was committed to serving China’s contemporary educational practice (Graesser et al., 2022). Gehlbach and Robinson (2021) claimed that the vigorous development of China’s education section had transported many excellent talents to society. Under globalization, the global competition among different nations has gradually changed into the soft power of talents, posing higher requirements for China’s education. Cinematography is an indispensable subject in China’s art system and a technology for the world’s common development. It was imperative to cultivate a group of high-quality cinematography talents. The teaching effect of the traditional cinematography was low due to the outdated teaching concept. Applying the theory of EPSY in cinematography teaching and innovating the traditional teaching concept would help improve the classroom teaching effect (Gehlbach and Robinson, 2021). Elmghaamez et al. (2022) contended that college Innovation and Entrepreneurship Education (IEE) aimed to cultivate college students’ innovation ability and employability. It was an educational model to improve the comprehensive quality of talents. United Nations Educational, Scientific, and Cultural Organization (UNESCO) believed that IEE could cultivate pioneering individuals in a broad sense. Innovation was the foundation of entrepreneurship, and there was an inseparable relationship between entrepreneurship and innovation (Elmghaamez et al., 2022). Putteeraj et al. (2022) pointed out that the employment of college students has become a hot issue widely concerned by the government, universities, and society under the world financial crisis. Using University Science and Technology Parks as platforms to build incubation and entrepreneurship bases and cultivate entrepreneurial talents could encourage college students to start businesses. This way, it played an important role in alleviating China’s employment pressure. It cultivated young people’s innovative spirit and promoted the transformation of scientific and technological achievements in higher institutions (Putteeraj et al., 2022). To sum up, the current cinematography education still lacks IEE. In particular, IEE is also a necessary measure to promote the common development of higher institutions and society. Therefore, researching and strengthening IEE in various disciplines in higher institutions is the main goal of the current education sector.

Thereupon, based on EPSY, this work first analyzes the current situation and characteristics of the teaching of Cinematography Major, then discusses the Cinematography Major and students’ creative practice strategies, and finally designs the research methods and basic ideas of the formulation of the teaching strategies of Cinematography Major from the perspective of EPSY. What is the innovation here? This work uses the knowledge of EPSY to investigate students in many aspects and combines the concept of IEE to make the teaching content of Cinematography Major more targeted and innovative. This work provides a reference for improving the teaching effect of Cinematography Major and the development direction of the film industry.

## Theory and Method

### Educational Psychology

Educational Psychology is a comprehensive subject that undertakes the teaching task of both psychology and pedagogy (Su, 2020). Education is an eternal social phenomenon. Since ancient times, education has always shouldered the important task of inheriting and developing human civilization. Early education mainly enables humans to master the skills of survival and the basic rules of human ecology through the transfer of skills in and between communities. In the educational process, human beings can quickly and correctly understand the outside world and promote human development through the reform of educational mechanisms during the educational process (Greig et al., 2019). The effectiveness of education is evaluated through a series of tests, and psychological research for educators and teachers is the most direct way to evaluate it (Patall, 2021). EPSY studies people’s psychological phenomena and combines psychology and education as a scientific discipline. Psychology generally divides the psychological content of the human body into two categories: psychological process and personality psychology. Among them, psychological process refers to the content of human psychological activities. The psychological process is divided into cognitive, emotional, and volitional processes. Through the cognitive process, humans understand their environments through the brain’s interaction with external stimuli (Glass and Kang, 2019). Cognition includes processes such as perception, memory, thinking, and imagination, which are human psychological activities against external environments. The most straightforward pedagogy teaches through cognition, so studying psychology in pedagogy becomes necessary to regulate the educational process through psychology (Prinz et al., 2021). The emotional process will come alongside cognition: human beings will exert certain psychological emotions or feelings about objective objects. In turn, man’s cognitive desire will be promoted by their emotions. Thus, the emotional process is also the internal driving force of the cognitive process. Through emotional promotion, human beings will recognize, develop, and transform the world (Drapeau, 2022). However, uncertain factors in the cognition process constitute cognition difficulties. Overcoming these hardships to achieve psychological goals becomes the volitional process that promotes the rapid development of human society (Zysberg and Schwabsky, 2021). The cognitive, emotional, and volitional processes are three independent processes in human psychology and have a certain correlation (Plucker and Makel, 2021). In particular, the cognitive process is the premise of the emotional process and the volitional process. People’s emotion generation through the cognition process depends on individual cognition degree. The volitional process plays a regulatory role between the cognitive and emotional processes. Therefore, pedagogy-oriented psychological (namely EPSY) research should consider the cognitive, emotional, and volitional processes to strengthen educational effects (Martínez et al., 2019).

**FIGURE 1:**
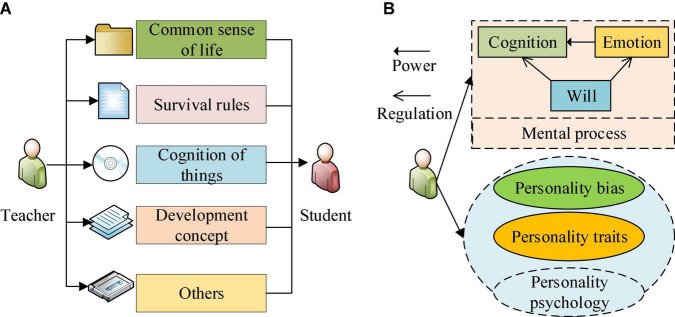
The main content and characteristics of Educational Psychology (EPSY). **(A)** The main content and characteristics of pedagogy, **(B)** The main content and characteristics of psychology.

Individual psychological qualities, acquired living environment, educational environment, and other differences all influence human psychological processes. This has given birth to personality psychology (Koenka et al., 2021) which mainly includes personality bias and personality characteristics. Personality bias or psychological bias involves dynamic psychological factors such as human needs, motivation, interests, ideals, and world outlook (Murjani and Nurjaman, 2022). By comparison, personality characteristics are relatively stable, genuinely reflecting the psychological characteristics of human beings, including temperament, ability, and personality. EPSY studies human psychological activities throughout the educational process. EPSY can explore human learning levels and psychological evolution. Meanwhile, EPSY analyzes pedagogy from the psychological perspective to formulate more appropriate teaching strategies (Han et al., 2020). The specific content and relationship between pedagogy and psychology are shown in Figure 1.

As shown in Figure 1, pedagogy provides a continuous impetus for human inheritance and development as the primary way of human knowledge dissemination. Psychology, as a reference, can provide feedback on the teaching process of pedagogy and analyze the specific role and achievements of education through psychological research. Therefore, EPSY, combing pedagogy and psychology, provides the driving force for pedagogy development and the research basis for psychology. EPSY is more specific and accurate than general psychology (Wardhani et al., 2022). The essence of psychology is scientifically explained in dialectical materialism. It is asserted that human psychology is an objective reflection of brain response, namely a brain function. Therefore, human psychological research can start with the brain, the organ where psychological activities are generated (Huang et al., 2019). Through psychological activities, humans understand the natural phenomena, mine the nature of things, and recognize the internal and external connections of things. Ultimately, they can reconstruct things that align with the facts through a complete psychological cycle (Torppa et al., 2020). Compared with EPSY, general psychology is relatively extensive but simple and studies the universal laws in people’s psychological activities and the common psychological activities. EPSY mainly studies students’ psychological activities and laws in the educational process, mainly aiming at the effect and quality of teaching, so it is more dedicated and profound (Colmar et al., 2019).

### Cinematography and Creative Practice Strategies of Students

Cinematography has entered the education industry of Chinese colleges since the 1950s, so a relatively mature state has been formed in the teaching process. However, with the development of the times, the educational problems of cinematography are also constantly emerging. The main training goal of cinematography is the combination of theory and creative orientation; that is, during the education process, students are required to master the theoretical knowledge of learning and need to have a certain creative orientation (Akser, 2020). As the main educational foundation of cinematography, theoretical cultivation is the main support for students’ creativity. The expressions of film art are diverse, but they take theoretical knowledge as the primary standard. A solid theoretical knowledge means a good mastery of the fundamental laws of film creation. Films reflecting a specific era can be created by teaching students the latest theoretical knowledge and production methods. In particular, the theoretical knowledge help students build the basic framework of film production and motivate students’ film creation. Film creation is a social activity that expresses and inherits various cultures, customs, and values in the form of film art. The way of film creation can establish correct values for the public and be a reference to society’s mainstream values (Croombs, 2019).

**FIGURE 2:**
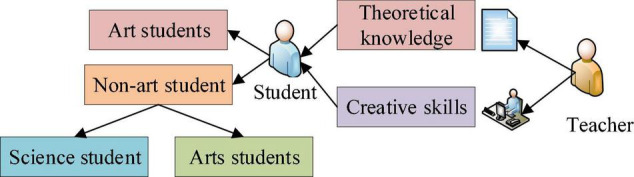
Student sources and teaching methods of Cinematography Major.

However, theoretical knowledge alone cannot serve to achieve the teaching goal; advanced creative technology and excellent artistic creation ability are also needed. Therefore, cinematography teaching has always been the mutual promotion and development of theoretical and practical creation (Szczepanik, 2021). The enrollment process for Cinematography Majors is also different from other majors. Cinematography Majors recruit both science students through the college entrance examination and art students before the exam (Suppia, 2020). Unlike many other majors, first-year students in Cinematography Majors do not have much film-related knowledge and skills. Teachers and students all have to start from scratch. Thus, it is necessary to impart theoretical knowledge while cultivating students’ film production expertise, namely, combining theoretical knowledge with creative practices (Marchetti, 2021). The composition of Cinematography Major students and the basic teaching methods are demonstrated in Figure 2.

Figure 2 indicates that Cinematography Major students come from different backgrounds. These students can roughly be classified by their family origin, gender, grade, and art skills (Krasowska et al., 2019). The school educational process strives to combine theory with practice. Then, students’ performance can be comprehensively analyzed by factoring in multiple influencing factors. In turn, the evaluation results can be used to devise targeted teaching and learning strategies for each student (Germini and Peltonen, 2021). As a result, Cinematography talents with comprehensive knowledge and quality can be cultivated (Hassan, 2021). Students’ knowledge mastery and creativity development might vary greatly due to the living environment, learning ability, and secondary-education level. Therefore, reasonable teaching strategies must be formulated in teaching theoretical knowledge and creative orientations according to the actual situation (Vallejo, 2020). For example, students with rich theoretical knowledge can be arranged with more practice and creativity fostering. Students with weak theoretical knowledge should be strengthened by intensive classroom teaching (Engelke, 2021). Therefore, in film industry development, it is necessary to continuously evaluate Cinematography Major students’ overall knowledge and skills according to the knowledge update and creative orientations innovation. Different teaching strategies can be implemented with the evaluation results to export more outstanding Cinematography talents and promote the film industry (Cuelenaere, 2020).

## The Teaching Model of Cinematography From the Perspective of Educational Psychology

**FIGURE 3:**
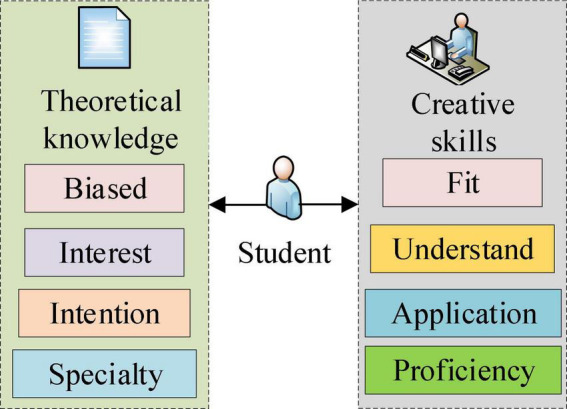
EPSY-based Cinematography teaching.

As mentioned above, Cinematography Majors educate students’ creative orientations based on theoretical knowledge. Theoretical knowledge and creative orientations promote each other and develop together. Therefore, customized teaching methods with graded-teaching intensities should be chosen according to the student’s learning ability and knowledge reserve. EPSY, focusing on studying students’ educational processes, plays a vital role in studying students’ teaching effects, learning ability, learning interests, and creative orientation. Accordingly, this work employs the EPSY theory to evaluate Cinematography Major students’ psychological status and analyze their film creation orientation and knowledge reserve. Specifically, students’ psychological cognition, psychological emotions, and psychological volition are analyzed to determine their learning state. The basic principle of EPSY-based analysis of Cinematography Major students’ knowledge reserve and creative orientation is given in Figure 3.

As Figure 3 illustrates, the pedagogy function module analyzes students’ psychological activities in the process of education through psychological methods by considering psychological cognition, psychological emotion, and psychological will.

Psychological cognition reflects students’ mastery of the theoretical knowledge of Cinematography, such as the history, purpose, and main technologies. By comparison, psychological emotion mirrors students’ interest in Cinematography, namely the learning motivation. Lastly, students’ mastery of Cinematography creative orientations is displayed through the psychological will. Further, entrepreneurial skills cannot simply be taught but require self-study and entrepreneurial practices. Thus, in EPSY-based Cinematography teaching, entrepreneurial skill fostering becomes a major challenge, essentially students’ problem-solving skills. In this regard, psychological cognition levels differ from student to student, which will lead to different Cinematography teaching effects. Therefore, Cinematography Major students must be evaluated through different classification indicators from the psychological perspective to analyze their learning abilities more refined. Based on this, more reasonable teaching methods can be implemented against students’ disparity development.

Following the above analysis, this work conducts a comprehensive study on Cinematography students by factoring in gender, grade, and artistic background through the Questionnaire Survey (QS) method. The research content has universal applicability, given that gender, grade, and major are the most basic and important indexes of current student classification. Table 1 lists the basic information (theoretical knowledge and creative orientations) of the 400 Cinematography Major students from the perspective of EPSY.

**TABLE 1:** Basic classification information of Cinematography Major students.

Standard	Classification	Number of people
Gender	Male	200
	Female	200
Grade	1	100
	2	100
	3	100
	4	100
Artistic background	Art students	200
	Non-art student	200

As shown in Table 1, this work studies the theoretical knowledge and creative orientations of Cinematography Major students using different classification indexes. The research results provide the primary reference for exploring new teaching strategies for Cinematography Majors. Apparently, there are an equal number of male and female students and evenly distributed students among the four grades. In terms of their artistic background, students are mainly divided into art students and non-art students, mainly referring to their high-school art education. Noticeably, the difference between art students and non-art students cannot be overlooked. Classifying students through their artistic backgrounds provides a more detailed reference basis for teaching Cinematography Majors. The students’ theoretical knowledge is mainly analyzed through the students’ cognition of the history, significance, purpose, basic norms, and materials of the Cinematography Major. The students’ creative orientations are mainly analyzed from multimedia equipment usability, video editing and synthesis, audio editing and synthesis, script creation, and video software manipulability. Here, 400 QS are distributed, recovering 398 valid ones, indicating a high validity rate of the QS. Finally, the QS results are comprehensively analyzed.

## Teaching Evaluation Results of Cinematography Majors From the Educational Psychology Perspective

### Evaluation of Gender Impact From the Educational Psychology Perspective

**FIGURE 4:**
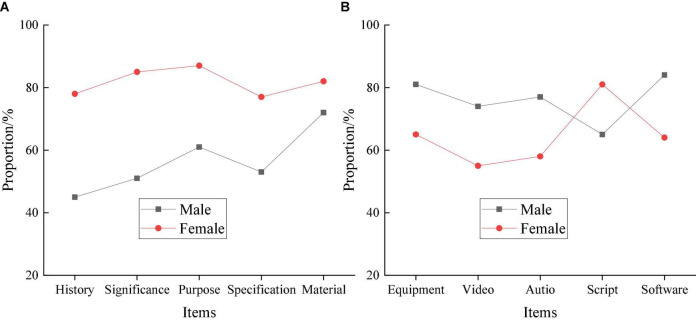
EPSY-based teaching effect evaluation of Cinematography Major through gender classification. **(A)** Theoretical knowledge. **(B)** Creative orientations.

The teaching effect of Cinematography Majors is evaluated from the EPSY perspective. Firstly, gender has a significant long-term impact on the teaching effect. Therefore, the teaching effect of Cinematography is analyzed through gender classification. Through gender analysis, male and female students can be educated at different levels to convey more Cinematography Major talents. The EPSY-based comprehensive evaluation results of the teaching effect gender classification are manifested in Figure 4.

In Figure 4, in the evaluation results of theoretical knowledge, girls’ overall performance is much better than that of boys. In particular, girls perform the best in the basic definition of Cinematography, with about 84% qualification rate, and perform the worst on the basic standard of Cinematography, with about 75% qualification rate. By comparison, boys perform the best in material cognition, with about a 74% qualification rate. They perform even worse in film history, with about a 46% qualification rate. Overall, boys and girls perform quite differently in theoretical knowledge evaluation: boys lag far behind girls. Thus, it is necessary to focus on cultivating theoretical knowledge for boys. In the evaluation results of creative orientations, the overall performance of boys is higher than that of girls. In particular, boys perform the best in video software manipulation, with about 84% qualification rate, and perform the worst in script creation, with about 65% qualification rate. In contrast, girls only beat boys in script creation in the creative orientations evaluation, with about an 80% qualification rate. The girls perform the worst in video editing and synthesis, with only about a 55% qualification rate. The numerical result indicates that the performance of boys and girls in theoretical knowledge and creative orientations is quite different. Girls are better than boys in theoretical knowledge, and boys are more proficient in creative orientations than girls.

### Evaluation of Grade Impact From the Educational Psychology Perspective

**FIGURE 5:**
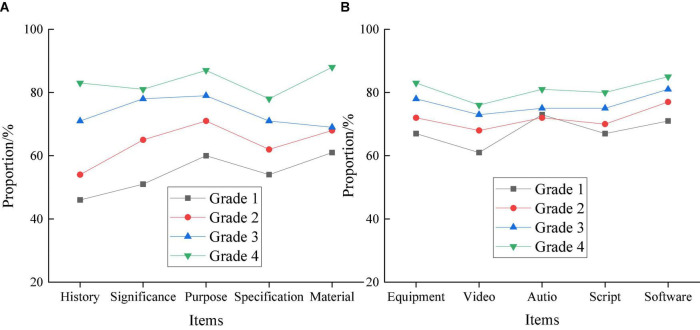
Evaluation results of different grades. **(A)** Theoretical knowledge. **(B)** Creative orientations.

Students’ learning process can be clearly understood by evaluating the teaching effect of students in Cinematography at different grades through EPSY. Students can be standardized to a certain extent by formulating corresponding plans to master the relevant knowledge of Cinematography. Meanwhile, the EPSY-based evaluation can provide a more accurate basis to improve learning and teaching effects. Figure 5 depicts the evaluation results of the teaching effect of students in different grades of Cinematography from the perspective of EPSY.

Apparently, the evaluation results of the theoretical knowledge of different grades vary greatly. Students’ score increases with the increase of the grade. First-grader’s score on the film history is the lowest, with about 45% qualification rate. Fourth-grader’s score on material cognition is the highest, with about 88% qualification rate. By comparison, in the test of creative orientations, the difference from grade to grade is relatively smaller than that of theoretical knowledge, and students’ scores will also improve with the increase in grades. In particular, the first-grader students’ score on video editing and synthesis is the lowest, with about a 60% qualification rate. Fourth-grade students’ score on the video software manipulation is the highest, with about 85% qualification rate. Thus, grade difference has less impact on students’ creative orientations.

### Evaluation of Artistic Background Impact From Educational Psychology Perspective

**FIGURE 6:**
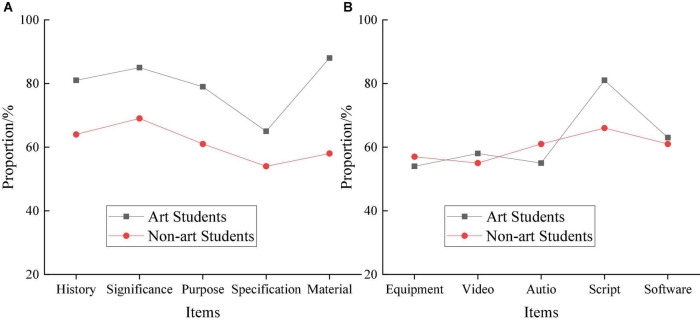
Evaluation results of students of different artistic backgrounds. **(A)** Art students. **(B)** on-art students.

Cinematography major students come from different artistic backgrounds, including art and non-art students and science and liberal arts students among non-art students. Therefore, evaluating the teaching effect of Cinematography through artistic background has a particular impact on the teaching mode and methods. The results of evaluating students through different artistic backgrounds are explained in Figure 6.

Obviously, there is a big gap between art students and non-art students in the theoretical knowledge test results. Specifically, art students perform the best in material cognition, with about 88% qualification rate, and perform the worst in the test of Cinematography norms, with about 65% qualification rate. Comparatively, non-art students perform the best in the basic definition of Cinematography, with about 70% qualification rate, and perform the worst in the test of Cinematography norms, with about 55% qualification rate. Meanwhile, in the test of creative orientations, the gap between art students and non-art students is tiny. The overall qualification rate is maintained at around 50–60% except for art students’ script creation which is about 80%. The finding implies that different artistic backgrounds greatly impact the teaching effect of students’ theoretical knowledge but have little impact on the teaching effect of students’ creative orientations.

### Evaluation of the Overall Impact on the Cinematography Teaching From the Educational Psychology Perspective

**FIGURE 7:**
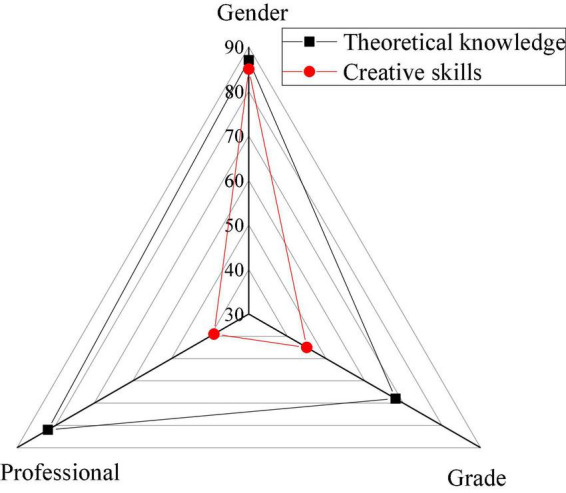
Overall EPSY-based evaluation of the Cinematography teaching effect.

The EPSY-based teaching evaluation can analyze the basic situation of Cinematography Major students and promote the reform of Cinematography. The EPSY-based overall evaluation results of the teaching effect of Cinematography are exhibited in Figure 7.

In Figure 7, the Cinematography teaching effect is evaluated from the aspects of gender, grade, and artistic background. Evidently, gender has the most profound impact on the teaching effect, which is closely related to psychological processes between different genders and psychological factors, such as personality characteristics. Thus, students’ learning can be strengthened through psychological counseling and guidance to promote the Cinematography teaching reform.

### Discussion

With the promotion of educational scale and depth in the overall society, talents’ innovation, and entrepreneurship skills have become a key metric for social development. Therefore, higher institutions and relevant majors implement teaching reforms to improve teaching quality and strengthen students’ innovation and entrepreneurship abilities. The aim is to cultivate more high-quality graduates for society. Besides, doing so improves social production ability and comprehensively promotes social development. In particular, the development of Cinematography plays an essential role in the film and television industry and is related to psychology. Therefore, comprehensively evaluating Cinematography Majors through psychology can improve the learning and teaching effect. It promotes the development of Cinematography and the art film and television industry. This work reveals that girls’ comprehensive performance is better than boys’ in theoretical knowledge evaluation. Girls perform best in the basic meaning of Cinematography, with about 84% qualification rate, and perform the worst in the Cinematography norms, with about 75% qualified. The male students perform the best in material cognition, with about 74% qualification rate, and perform the worst in film history, with about 46% qualification rate. Overall, there are significant differences between boys and girls in theoretical knowledge reserve. The overall performance of boys lags behind girls. Hence, there is a need to focus on cultivating boys’ theoretical knowledge. On the other hand, in the evaluation results of creative orientations, the all-around performance of boys is higher than that of girls. Boys perform best in video software manipulation, with about 84% qualification rate, and perform the worst in scriptwriting, with about 65% qualification rate. By comparison, girls beat boys in script creation in the creative orientation test, with about 80% qualification rate, and have performed the worst in video editing and composition, with about 55% qualification rate. Thus, there are significant differences between boys and girls in theoretical knowledge and creative orientation. Girls have better theoretical knowledge than boys, and boys have more skilled creative orientations than girls. Meanwhile, the test results of theoretical knowledge differ greatly from grade to grade, and the difference increases with grades. The first-grade Cinematography Majors has the lowest score in film history with about a 45% qualification rate. The fourth grade has the highest score on the theoretical cognition test, with about 88% qualification rate. In the creative orientation test, the difference between grades is smaller than the difference in theoretical knowledge. The test score increases with students’ grades. Grade one has scored the lowest on the video editing and composition test, with about a 60% qualification rate. Comparatively, grade four has the highest score in video software tests, with about an 85% qualification rate. There is little difference in creative orientation between different grades. Lastly, the evaluation of students from different artistic backgrounds reflects a large gap between art students and non-art students in theoretical knowledge reserve. Art students perform best in material cognition, with about 88% qualification rate, and perform the worst in the Cinematography norm test, with about 65% qualification rate. Non-art students have the best scores in the basic definition of Cinematography, with about 70% qualification rate, and perform the worst in the Cinematography norms test, with about 55% qualification rate. In the creative orientation test, the gap between art students and non-art students is not large. The overall qualified rate is about 50–60%, except for the qualification rate of art students in script creation (about 80%). Therefore, different artistic backgrounds greatly impact the teaching effect of students’ theoretical knowledge but have little impact on the teaching effect of students’ creative orientations. Comprehensive research shows that gender has the most profound influence on the teaching effect, which is related to psychological factors such as psychological processes and personality characteristics between different genders. Finally, through psychological guidance, teachers can comprehensively strengthen students’ learning effect, innovation, and entrepreneurship ability and promote the teaching reform of Cinematography Majors.

## Conclusion

With the development of society, innovation and entrepreneurship theory have become the main direction of higher education. Therefore, promoting IEE in higher institutions through research plays a vital role in developing the education industry. Based on this, this work first evaluates the innovation and entrepreneurship teaching effect of Cinematography Major from the perspective of EPSY. Then, it makes targeted teaching plans and strategies to improve Cinematography Majors’ teaching effect and teaching system. The results show a large gap in the teaching effect among boys and girls. Girls’ theoretical knowledge is better than boys; boys’ creative orientations are superior to girls. Meanwhile, there are significant differences in theoretical knowledge between different grades, which shows that the teaching results of the school are significant. At the same time, in terms of creative orientations, there are small differences in different grades, which shows that the practical effect of students is not satisfactory. Finally, from students’ artistic backgrounds, art students and non-art students have significant differences in theoretical knowledge but little differences in creative orientations. Although this work has carried out many aspects of teaching evaluation, it is not detailed enough in influencing factor analysis. Therefore, future work will study the teaching effect of Cinematography Major through more refined influencing factors.

## Data Availability Statement

The raw data supporting the conclusions of this article will be made available by the authors, without undue reservation.

## Ethics Statement

The studies involving human participants were reviewed and approved by Communication University of China Ethics Committee. The patients/participants provided their written informed consent to participate in this study. Written informed consent was obtained from the individual(s) for the publication of any potentially identifiable images or data included in this article.

## Author Contributions

The author confirms being the sole contributor of this work and has approved it for publication.

## Conflict of Interest

The author declares that the research was conducted in the absence of any commercial or financial relationships that could be construed as a potential conflict of interest.

## Publisher’s Note

All claims expressed in this article are solely those of the authors and do not necessarily represent those of their affiliated organizations, or those of the publisher, the editors and the reviewers. Any product that may be evaluated in this article, or claim that may be made by its manufacturer, is not guaranteed or endorsed by the publisher.

## Appendix

### Questionnaire Survey

1. Basic information:

A. What’s your gender? ◻ Male ◻ Female

B. What grade are you in?

◻ Grade one ◻ Grade two ◻ Grade three ◻ Grade four

C. Do you have a professional artistic education back in high school?

◻ No ◻ Yes

2. Theoretical knowledge of Cinematography:

A. Please talk about the history of Cinematography:

B. Please talk about the teaching significance of Cinematography:

C. Would you like to talk about the teaching purpose of Cinematography Majors?

D. Please talk about the basic norms of Cinematography:

E. Please briefly analyze the following materials:

Using the specific tasks and works in the history of Chinese literature, talk about the relationship between Cinematography and innovation and entrepreneurship.

3. Professional creative orientations of Cinematography:

A. How proficient are you in using multimedia equipment (1–5 denote “completely diskilled, can operate simply, unskilled, proficient, and very proficient”)?

◻ 1 ◻ 2 ◻ 3 ◻ 4 ◻ 5

B. How proficient are you in video editing and composition (1–5 denote “completely diskilled, can operate simply, unskilled, proficient, and very proficient”)?

◻ 1 ◻ 2 ◻ 3 ◻ 4 ◻ 5

C. How proficient are you in audio editing and composition (1–5 denote “completely diskilled, can operate simply, unskilled, proficient, and very proficient”)?

◻ 1 ◻ 2 ◻ 3 ◻ 4 ◻ 5

D. How skilled are you in script writing (1–5 denote “completely diskilled, can operate simply, unskilled, proficient, and very proficient”)?

◻ 1 ◻ 2 ◻ 3 ◻ 4 ◻ 5
